# Sleep Quality, Glycemic Control, and Daytime Dysfunction in Type 2 Diabetes

**DOI:** 10.3390/healthcare14070838

**Published:** 2026-03-25

**Authors:** Ceren Gür, Seda Şenkardeş Kandemir

**Affiliations:** Department of Internal Medicine, Bağcılar Training and Research Hospital, University of Health Sciences, Istanbul 34200, Türkiye; seda_senkardes@hotmail.com

**Keywords:** type 2 diabetes mellitus (T2DM), metabolic dysregulation, daytime sleepiness and dysfunction, sleep quality, Pittsburgh Sleep Quality Index (PSQI)

## Abstract

**Background and Objectives:** Adults diagnosed with type 2 diabetes mellitus (T2DM) frequently exhibit diminished sleep quality, which is affected by their glycemic control. Both poor sleep and inadequate glycemic management increase the risk of complications worldwide. However, the relationship between sleep quality, daytime dysfunction, and glycemic control in adults with T2DM is not well understood. To address this gap, this study aimed to examine the association between overall sleep quality, including daytime dysfunction, and glycemic control in adults with T2DM. **Materials and Methods:** A hospital-based cross-sectional study included 200 T2DM patients (103 females, 97 males) from January 2019 to January 2020. The Pittsburgh Sleep Quality Index (PSQI) was administered to each participant to assess sleep quality, including daytime dysfunction. Glycemic control was assessed by measuring hemoglobin A1c (HbA1c) and fasting blood glucose (FBG) levels. The associations among sleep quality, daytime dysfunction, and glycemic metrics were examined utilizing both conventional statistical methods and Bayesian analytical approaches. **Results:** A total of 32% of patients had poor sleep quality (PSQI > 5), and 43.5% reported daytime dysfunction. Higher HbA1c and fasting blood glucose were each correlated with longer sleep-onset latency and greater daytime dysfunction. In multivariable analyses, higher HbA1c and longer sleep-onset latency were independently associated with poor sleep quality (generalized linear model, R^2^ = 0.602), whereas higher fasting blood glucose and longer sleep-onset latency were associated with greater daytime dysfunction severity (R^2^ = 0.378). **Conclusions:** Sleep quality and delay in falling asleep are interdependent with impaired glycemic control and daytime dysfunction in T2DM. Future randomized trials targeting sleep-onset latency are warranted to determine causal effects on glycemic outcomes.

## 1. Introduction

Type 2 diabetes mellitus (T2DM) is a growing global health problem, affecting millions and increasing the risk of cardiovascular, kidney, and nerve issues [1]. Managing T2DM is complicated because of various complications caused by blood sugar fluctuations, blood vessel problems, and nerve degeneration, all of which lower patients’ quality of life and put pressure on healthcare systems [2].

Recent research shows that sleep disturbances both result from and contribute to poor glycemic control. Approximately 30% of adults with diabetes experience excessive daytime sleepiness [3]. Insufficient sleep in T2DM can worsen glycemic control through mechanisms involving insulin resistance [4], changes in appetite hormones (leptin, ghrelin) [5], inflammation [6], and sympathetic activation that promotes insulin resistance [4]. Sleep problems and metabolic disorders can reinforce each other, creating a harmful cycle influenced by stress, disrupted routines, mental health issues, and medical conditions such as cardio-metabolic or endocrine diseases [7,8]. These mechanistic links are also supported by recent systematic reviews and meta-analyses showing that disturbed sleep is associated with insulin resistance, altered appetite regulation, inflammation, and poorer glycemic outcomes in T2DM [9,10,11].

Sleep disorders, including short or irregular sleep duration, poor sleep quality, insomnia, obstructive sleep apnea (OSA), and excessive daytime sleepiness (EDS), are common in adults and negatively affect cognition, mood, and daily functioning [12]. Sleep quality is a complex concept. The Pittsburgh Sleep Quality Index (PSQI) measures subjective sleep quality, latency, duration, efficiency, disturbances, sleep medication use, and daytime dysfunction; and PSQI/component-7 involves impaired daytime functioning, such as difficulty staying awake and reduced energy. Daytime dysfunction and decreased efficiency are linked to higher hemoglobin A1c (HbA1c), fatigue, poor self-care, and lower adherence to diabetes management [13,14]. Notably, patients with poor glycemic control experience more sleep disturbances than those with optimal control [15]. Recognizing these connections can guide the development of targeted interventions to improve sleep health and diabetes outcomes.

This study aims to explore the connections between sleep quality, daytime dysfunction, and glycemic control in adults with T2DM. It also examines which specific PSQI subdimensions are most strongly linked to higher HbA1c levels. Unlike most previous research that focuses on overall sleep quality, this study enhances the literature by specifically analyzing PSQI subdimensions, especially sleep-onset latency and daytime dysfunction, in relation to glycemic control in adults with T2DM.

## 2. Materials and Methods

### 2.1. Study Design, Setting, and Participants

This single-center, cross-sectional study consecutively enrolled 200 adults (103 females, 97 males) from the outpatient clinic of Bağcılar Training and Research Hospital, University of Health Sciences, Istanbul, Türkiye, between 1 January 2019 and 1 January 2020. The inclusion criteria were having type 2 diabetes, being >18 years old, and having the cognitive ability to complete the questionnaires (PSQI, [16]) and provide informed consent. Individuals with severe psychiatric conditions, diagnosed sleep disorders, or those taking sedatives or steroid medications were not included in the study.

### 2.2. Measures and Data Collection

Glycemic indices included fasting blood glucose (FBG) and HbA1c. Following an overnight fast (≥8 h), venous blood samples were obtained during routine clinic visits and analyzed by the hospital’s central laboratory according to standardized clinical protocols. Serum samples were analyzed using the Roche–Hitachi Cobas 8000 autoanalyzer (Mannheim, Germany) for measurements including FBG and HbA1c.

Subjective sleep quality was assessed using the Pittsburgh Sleep Quality Index (PSQI) [16], a validated self-report questionnaire that evaluates sleep and disturbances over a one-month period. The PSQI was chosen for its reliability, standardization, and appropriateness for the hospital-based cross-sectional design, while objective measures such as actigraphy were not used. The index includes 19 scored self-report questions and 5 unscored questions for bed partners, covering seven domains: (1) subjective sleep quality, (2) latency, (3) duration, (4) efficiency, (5) disturbances, (6) medication use, and (7) daytime dysfunction. Each domain is scored from 0 (no difficulty) to ≥2 (severe difficulty), with the total score ranging from 0 to 21; scores above 5 suggest poor sleep quality, while scores of 5 or below indicate good sleep quality.

### 2.3. Ethical Considerations

The protocol was reviewed and approved by the Institutional Ethics Committee of Bağcılar Training and Research Hospital (Approval Number: 2019.01.2.03.120.r1.007). The study adhered to the Declaration of Helsinki and local regulatory requirements. All participants provided written informed consent before data collection.

### 2.4. Statistical Analysis

Normality was assessed using the Kolmogorov–Smirnov test, inspection of skewness/kurtosis, and Q–Q plots. Data are presented as mean ± standard deviation (SD), median (interquartile range, IQR), or n (%), as appropriate. Bivariate associations were examined using Bayesian Kendall’s τ (for ordinal/non-normal data) and Pearson correlation (r value, for normally distributed variables). Additionally, the H0 (independence) and H1 (association) hypotheses were determined, the data were subjected to Bayesian calculations, and the evidence was presented through the Bayes factor (BF_10_). Bayesian analysis was used alongside conventional statistical methods to supplement *p*-value-based inference by measuring the strength of evidence supporting the alternative over the null hypothesis, especially for ordinal and non-normally distributed variables. BF_10_ values were interpreted as indicating evidence in favor of the alternative hypothesis, with values > 1 supporting H1 and values < 1 supporting H0; conventionally, values between 1 and 3 were considered weak/anecdotal evidence, 3–10 moderate evidence, 10–30 strong evidence, 30–100 very strong evidence, and >100 extreme evidence. The stretched beta prior width was set to =1. Non-normal data were transformed into an approximate distribution before being subjected to the Pearson test. A Generalized Linear Model was used as a multivariate model to evaluate possible confounding factors and correlation strength (R^2^). The estimated marginal means table and graph are presented. Effect sizes are reported as odds ratios (OR) with 95% confidence intervals (CI). All calculations were performed using Jamovi v2.3.18 statistical package program with the jsq and GAMLj extensions.

A post hoc power analysis was performed using G*Power software (ver. 3.1.9.7) based on the primary outcome of the study, namely the relationship between sleep quality and glycemic control in patients with T2DM [17]. Using a two-tailed correlation model with an assumed effect size of 0.20, an α error probability of 0.05, and a total sample size of 200, the achieved power was 0.81. Thus, the final sample size was deemed sufficient for the planned analyses.

## 3. Results

The cohort comprised 200 adults with T2DM (103 females, 51.5%; 97 males, 48.5%) with a mean age of 59.6 ± 9.3 years. According to the PSQI global score, 32% of patients (n = 64) reported poor subjective sleep quality, whereas 68% (n = 136) reported good sleep quality. Daytime dysfunction, a PSQI component, was absent in 43.5% of participants (n = 87), reported as mild in 33.0% (n = 66), and severe in 23.5% (n = 47). A summary of data for glycemic control parameters, HbA1c and FBG in relation to sleep quality categories is presented in Table 1.

Bayesian correlations were conducted to examine associations of subjective sleep quality and daytime dysfunction with HbA1c, FBG, PSQI global score, sleep latency, sleep duration, and biological sex. There was moderate evidence that sleep duration and gender were independent of both sleep quality and daytime dysfunction (BF_10_ < 0.33; Table 2). In contrast, HbA1c, FBG, PSQI global score, and sleep latency each showed clear, positive associations with subjective sleep quality and daytime dysfunction, with Bayes factors indicating extreme evidence for the alternative hypothesis (BF_10_ > 100; Table 2). Notably, sleep latency correlated with higher HbA1c (r = 0.222; BF_10_ = 11.81) and higher FBG (r = 0.220; BF_10_ = 10.83), representing small-to-moderate effect sizes supported by strong Bayesian evidence. Collectively, these results suggest that while sleep duration and sex do not meaningfully relate to the targeted sleep outcomes, glycemic indices (HbA1c, FBG) and core PSQI score, particularly sleep latency, are meaningfully linked to both subjective sleep quality and daytime dysfunction.

**Figure 1:**
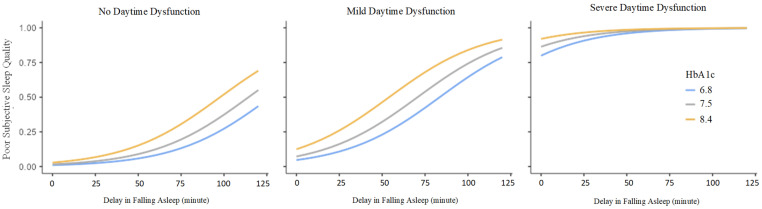
Model-based estimated probability of poor subjective sleep quality according to delay in falling asleep and HbA1c (%) stratified by daytime dysfunction severity. Panels represent no, mild, and severe daytime dysfunction. Within each panel, lines indicate HbA1c levels of 6.8%, 7.5%, and 8.4%. The y-axis shows the estimated probability of poor subjective sleep quality derived from the generalized linear model.

A multivariate GLM analysis was used to assess HbA1c, FBG, sleep latency, and daytime dysfunction as confounders of sleep quality. FBG was not significant. Higher HbA1c (48.0% at 8.4%), longer sleep latency (40.8% at 30 min), and severe daytime dysfunction (94.9%) were linked to increased poor sleep quality (Table 3). Figure 1 illustrates these associations across HbA1c and sleep latency, stratified by daytime dysfunction. In the right panel (severe dysfunction), higher HbA1c levels (8.4%) are associated with a sharper rise in poor sleep quality as sleep latency increases, whereas the increase is less pronounced at 6.8%, with 7.5% showing intermediate results. Among those with mild or no daytime dysfunction, differences in HbA1c levels are more pronounced across latency, indicating an association between higher HbA1c and poorer sleep quality when daytime dysfunction is minimal. As shown in the estimated marginal means table (Table 3) and graph (Figure 1), each 1-unit (approximately 1%-point) increase in HbA1c levels was associated with a 1.94-fold increase in the odds of reporting poor sleep quality (95% CI: 1.29–3.02), while each 1 min delay in falling asleep increased this likelihood by 1.04 times (95% CI: 1.01–1.08). Daytime dysfunction was strongly linked to poor sleep: mild dysfunction raised the odds by 4.83 times (95% CI: 1.36–22.88), and severe dysfunction increased risk by 391 times (95% CI: 72–3685). Overall, HbA1c, delayed sleep onset, and daytime dysfunction were linked to poor sleep quality (GLM, R^2^ = 0.602).

**Figure 2:**
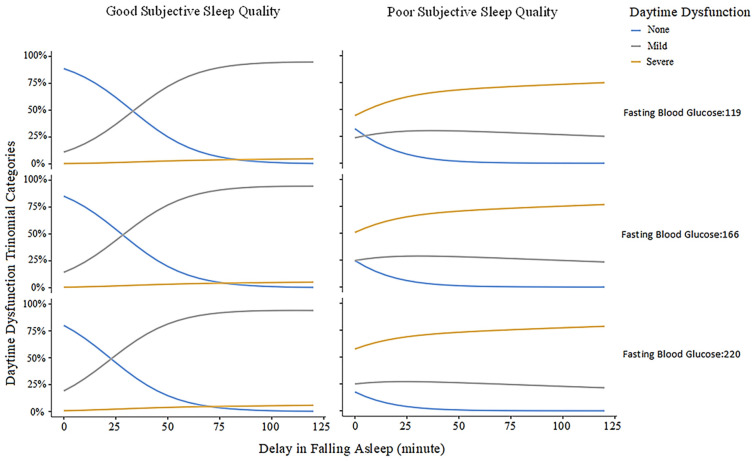
Model-based estimated probabilities of daytime dysfunction categories based on delay in falling asleep (minutes) and fasting blood glucose (FBG, mg/dL), grouped by subjective sleep quality. Each panel shows lines representing the estimated probabilities of no, mild, and severe daytime dysfunction from the generalized linear model.

In GLM predicting daytime dysfunction, longer sleep-onset latency, higher FBG, and poorer subjective sleep quality were associated with greater dysfunction severity. Estimated marginal probabilities (Table 4) show that poorer sleep quality is more likely with longer latency and greater dysfunction: at 30 min, severe dysfunction occurs in 35.2% versus 23.2% with no dysfunction; at FBG = 220 mg/dL, mild dysfunction is seen in 40.3% versus 24.0% with no dysfunction. Severe dysfunction is much more common with poor sleep quality than good (67.1% vs. 2.0%). Figure 2 shows that longer sleep-onset latency is associated with greater daytime dysfunction, with effects moderated by subjective sleep quality and FBG (119, 166, and 220 mg/dL). Among those with good sleep quality, dysfunction rises gradually, more so at higher FBG (166 and 220 mg/dL) than at 119 mg/dL. In contrast, poor sleep quality amplifies this relationship, yielding a steeper increase in dysfunction as latency lengthens, particularly at elevated FBG. As shown in the estimated marginal means table (Table 4) and graph (Figure 2), participants with mild dysfunction had a 1.06-fold higher risk of longer sleep latency per minute compared to those without dysfunction (95% CI: 1.03–1.10). Each 10 mg/dL increase in FBG raised the odds of mild dysfunction by 1.07 times (95% CI: 0.41–6.74), and poor sleep quality increased this likelihood by 5.95 times (95% CI: 1.48–3.91). Participants with severe daytime dysfunction were 1.07 times more likely to report each extra minute of sleep latency (95% CI: 1.03–1.11), and every 10 mg/dL increase in FBG increased risk by 1.09 times (95% CI: 1.02–1.15). Notably, poor sleep quality increased the odds of severe daytime dysfunction by 354 times (95% CI: 54–2331). Overall, daytime dysfunction was associated with prolonged sleep-onset latency, elevated fasting blood glucose levels, and reduced sleep quality (GLM, R^2^ = 0.378).

## 4. Discussion

This cross-sectional, single-center cohort analysis of adults with type 2 Diabetes Mellitus (T2DM) revealed that impaired subjective sleep quality and pronounced daytime dysfunction were robustly correlated with adverse glycemic indices, particularly elevated Hemoglobin A1c (HbA1c) and fasting blood glucose (FBG) concentrations. These associations were paralleled by higher Pittsburgh Sleep Quality Index (PSQI), with sleep-onset latency emerging as a key contributor. Conversely, self-reported sleep duration and sex did not yield significant associations with the sleep outcomes assessed. These results refine the existing literature by specifying which components of sleep health most directly correspond to glycemic control, underscoring latency and daytime dysfunction as potential focal points for clinical intervention. The overall pattern is supported by some clinical cohort studies showing the prevalence of poor sleep quality in patients with T2DM [18,19,20,21,22,23] and underscores that a substantial fraction of adults with T2DM experience sleep-related impairment. The main contribution of this study is that it emphasizes overall sleep measures by focusing on specific PSQI subdimensions, especially sleep-onset latency and daytime dysfunction, as important clinical factors related to glycemic control in adults with T2DM.

In this cohort study, falling asleep latency correlated with both HbA1c and FBG, which is consistent with a bidirectional association between sleep and glycemic control. There are various pathophysiological mechanisms underlying the disruption of falling asleep that may be related to hyperglycemia. Among the contributing factors, nocturnal hyperglycemia is particularly salient, as it may disrupt sleep continuity by provoking increased nocturnal urination, manifesting as polyuria and nocturia. Furthermore, prolonged nocturnal growth hormone secretion and elevated inflammatory markers in response to sleep restriction have been linked to insulin [13]. Circadian misalignment worsens glucose tolerance and cardiometabolic physiology even independent of time-in-bed [24,25]. Within this framework, experimental sleep restriction elicits insulin resistance and sympathetic activation, whereas poor sleep quality modifies appetite-regulating hormones toward energy balance (reduced leptin, increased ghrelin) and provokes chronic low-grade inflammation that compromises insulin signaling [4,5,6,26,27]. Additionally, prospective data highlighting the primacy of sleep quality relative to duration in predicting T2DM onset [20] reinforce the relevance of latency and continuity rather than sleep quantity alone. Another fact is that obstructive sleep apnea (OSA) syndrome might exacerbate sleep disturbance in T2DM patients [28]. Accordingly, nocturnal symptoms (polyuria/nocturia, neuropathic discomfort) and comorbid OSA may fragment sleep and prolong latency, sustaining hyperglycemia via inflammatory–autonomic dysregulation. OSA may also be a confounder, as it is independently associated with both impaired sleep quality and poor glycemic control. Therefore, the absence of formal OSA assessment in this study should be considered when interpreting the observed associations.

Daytime dysfunction was more strongly correlated with elevated FBG than with HbA1c, suggesting that acute blood glucose fluctuations may be more closely associated with daytime functional impairment than chronic glycemic control. In interpreting these results, it is crucial to distinguish between FBG, which more accurately reflects immediate glycemic status, and HbA1c, an indicator of long-term glycemic control. The observed stronger correlation between daytime dysfunction and FBG may suggest a more direct link to acute glycemic fluctuations rather than extended glycemic exposure. Previous studies have reported that poor glycemic control can lead to decreased attention, fatigue, and reduced performance in daily activities [29,30,31]. Notably, longer sleep-onset latency was associated with glycemic parameters and emerged as an independent correlation of both poor sleep quality and daytime dysfunction. Each minute increase in sleep delay was associated with a 6% higher likelihood of daytime dysfunction. Clinically, even a modest 10–15 min reduction in latency may be associated with a lower probability of daytime dysfunction, providing a concrete counseling target for routine diabetes visits. This observation aligns with literature showing that longer sleep-onset latency can disrupt sleep continuity and circadian rhythms, leading to inattention, fatigue, and decreased daytime function [32]. Taken together, our findings suggest that even modest improvements in sleep initiation (e.g., a 10–15 min reduction in latency) could meaningfully shift the probability of daytime dysfunction, a point that can be translated into practical counseling and shared goal-setting. Daytime dysfunction was also strongly linked to poor sleep quality in the multivariable analysis. However, the wide confidence intervals, especially for the severe dysfunction category, suggest considerable uncertainty about the strength of this link. As a result, these estimates should be viewed with caution and verified in larger studies. Additionally, the very high odds ratios may partly result from sparse data or limited model stability in some categories, making interpretation difficult. Future research with larger samples or different category setups may help clarify these findings.

Another point is that self-reported sleep duration and biological sex were not informative predictors in this cohort. Although prior meta-analyses have linked both short and long sleep with incident T2DM [33], and some studies report worse subjective sleep in women [34], our findings suggest that differences in sleep quality cannot be attributed solely to biological factors related to gender. Nevertheless, this result warrants careful interpretation, as sex-related differences in sleep patterns can vary across populations and may be influenced by undetected clinical or psychosocial factors. Regarding sleep duration, most participants in our study reported sleeping 6–8 h, yielding a relatively narrow range that may have limited our ability to detect significant associations. Additionally, because sleep duration was self-reported, some measurement-related inaccuracies cannot be ruled out [35]. Therefore, screening approaches restricted to duration are less informative than concise, targeted evaluations emphasizing latency, efficiency, and daytime performance.

Our results corroborate prior epidemiological and clinical evidence linking poor sleep quality to increased T2DM risk and more complex disease management. Systematic review data confirm poor sleep quality as a risk factor for T2DM development [10,36], while cross-sectional and cohort studies consistently associate reduced sleep quality with higher HbA1c [37]. Within a Turkish sample, we refine these observations by showing that PSQI subdomains, particularly sleep latency, are distinctly associated with glycemia and daytime functioning. Given the high prevalence of excessive daytime sleepiness (>30%) [3] and insomnia-type symptoms [12] among individuals with diabetes, such subdomain-specific findings provide valuable guidance for targeted screening and intervention strategies.

This study has some limitations. This study’s single-center, cross-sectional design limits the generalizability of its findings. The results are from a single hospital and may not apply to other populations or clinical settings; thus, they may not establish cause-and-effect relationships or monitor changes over time. These limitations should be considered when interpreting the findings. Objective sleep measures (actigraphy/polysomnography) and circadian phase markers were unavailable, and sleep assessment relied on self-reported PSQI data without objective validation. Formal OSA screening was also not conducted, and other potentially relevant comorbidities were not systematically assessed, which may have led to residual confounding. Moreover, important clinical variables such as body mass index (BMI), diabetes duration, and treatment characteristics were not systematically collected, limiting the ability to adjust for potential confounding. Additionally, nocturia/nocturnal polyuria, a possible mediator of the latency–glycemia relationship, was not measured. Despite these limitations, the study demonstrates several notable strengths: (i) the application of subdomain-level sleep phenotyping, which identifies latency and daytime dysfunction as clinically salient targets; (ii) the integration of Bayesian inference, GLM-based marginal estimates, and probability visualizations to ensure methodological robustness; and (iii) concordance with mechanistic and clinical literature addressing hyperglycemia-related nocturnal symptoms, hyperarousal and inflammation, circadian misalignment, and OSA. Notably, the stronger association of daytime dysfunction with FBG levels relative to HbA1c highlights the clinical relevance of addressing short-term glycemic variability in parallel with nocturnal sleep initiation and consolidation.

Future research should prioritize longitudinal and randomized controlled trials to determine whether interventions aimed at improving sleep initiation and continuity—such as cognitive behavioral therapy for insomnia (CBT-I), circadian light timing, or structured exercise scheduling—yield sustained improvements in HbA1c independent of weight change and to evaluate subgroup-specific effects. Pragmatic, clinic-based programs that integrate brief sleep screening focused on latency, efficiency, and daytime functioning with medication optimization and self-management support appear particularly promising. Moreover, from a clinical perspective, these findings support including brief sleep-related questions in routine diabetes appointments, especially regarding sleep-onset latency, daytime dysfunction, and potential symptoms of sleep-disordered breathing. Such screening can help identify patients who may benefit from sleep-focused counseling or further assessment as part of comprehensive diabetes management. Given the high prevalence of excessive daytime sleepiness and its established associations with glycemic regulation and treatment adherence, strategies that simultaneously address nocturnal sleep and daytime functioning are likely to confer the greatest benefit. In adults with T2DM, poor sleep quality—especially prolonged sleep-onset latency—emerges as a salient correlate of impaired glycemic control and daytime dysfunction. Accordingly, subgroup analyses are needed to identify patient populations most likely to benefit, thereby informing tailored interventions. Routine, structured assessment of sleep parameters should be incorporated into diabetes care, and interventions that reduce latency and stabilize nocturnal glycemia may enhance both daytime functioning and long-term disease management. Rigorous two-arm trials comparing targeted treatments for delayed sleep onset with usual care are essential to clarify causal pathways and optimize outcomes.

## 5. Conclusions

This study revealed that poor sleep quality and longer time to fall asleep are linked to worse glycemic control and daytime dysfunction in patients with T2DM. These findings suggest that routinely assessing sleep quality, especially sleep-onset latency and daytime functioning, could provide valuable clinical insights for diabetes management. However, these results should be interpreted with caution due to the study’s limitations, such as its cross-sectional, single-center design, reliance on self-reported sleep data, and absence of formal assessment of comorbid sleep conditions like OSA. Incorporating sleep assessment into standard diabetes care is crucial. Future well-designed randomized controlled trials comparing targeted treatment for delayed sleep-onset with usual care, along with longitudinal HbA1c and FBG measurements, are needed to establish causal relationships and determine if improving sleep can improve metabolic and functional outcomes.

## Figures and Tables

**Table 1:** Descriptive status of the patient group.

	Median (IQR)	Mean ± SD
HbA1c (%)	7.5 (6.8–8.4)	7.7 ± 1.4
FBG (mg/dL)	166 (119–220)	188.9 ± 94.1
PSQI score	6 (4–8)	6.6 ± 2.7

HbA1c, hemoglobin A1c; FBG, fasting blood glucose; PSQI, Pittsburgh Sleep Quality Index; IQR, interquartile range; SD, standard deviation.

**Table 2:** Correlations between sleep quality and daytime dysfunction.

	Subjective Sleep Quality		Daytime Dysfunction	
	r	BF_10_	r	BF_10_
Daytime dysfunction	−0.677	>100	-	-
HbA1c	−0.313	>100	0.337	>100
Fasting blood glucose	−0.330	>100	0.375	>100
PSQI total	−0.609	>100	0.615	>100
Delay in falling asleep	−0.255	>100	0.328	>100
Sleep duration	−0.035	0.121	0.069	0.259
Biological sex	−0.042	0.137	0.045	0.145
Age	0.038	0.127	−0.037	0.125

HbA1c, hemoglobin A1c; PSQI, Pittsburgh Sleep Quality Index; BF_10_, Bayes factor in favor of the alternative hypothesis. Correlations were examined using Pearson correlation or Bayesian Kendall’s τ, as appropriate.

**Table 3:** Estimated marginal means table of subjective poor sleep quality.

		Poor Sleep Quality OR (95% CI)
HbA1c (%)	6.8	24.2% (12.4–41.8)
	7.5	33.7% (20.3–50.3)
	8.4	48.0% (31.4–65.1)
Delay in falling asleep (min)	10	25.1% (13.0–43.0)
	20	32.5% (19.6–48.7)
	30	40.8% (25.5–58.1)
Daytime Dysfunction	None	4.58% (1.4–13.9)
	Mild	18.8% (10.7–30.9)
	Severe	94.9% (80.8–98.8)

HbA1c, hemoglobin A1c; OR, odds ratio; CI, confidence interval. Estimated marginal means were derived from the generalized linear model (GLM).

**Table 4:** Estimated marginal means table of daytime dysfunction possibility.

		Daytime Dysfunction	OR (95% CI)
Delay in falling asleep (min)	10	None	42.4% (31.6–53.2)
		Mild	26.8% (15.4–38.1)
		Severe	30.8% (20.1–41.5)
	20	None	32.6% (25.3–39.9)
		Mild	34.0% (24.0–44.0)
		Severe	33.4% (24.5–42.2)
	30	None	23.2% (14.9–31.6)
		Mild	41.5% (31.0–52.1)
		Severe	35.2% (27.3–43.2)
Fasting blood glucose (mg/dL)	119	None	34.4% (24.5–44.3)
		Mild	33.9% (21.2–46.7)
		Severe	31.7% (20.3–43.1)
	166	None	29.4% (21.7–37.1)
		Mild	36.9% (26.4–47.5)
		Severe	33.6% (24.6–42.7)
	220	None	24.0% (15.9–32.1)
		Mild	40.3% (30.1–50.5)
		Severe	35.7% (28.1–43.2)
Subjective sleep quality	Good	None	49.4% (36.8–62.0)
		Mild	48.7% (36.2–61.2)
		Severe	2.0% (0–5.1)
	Poor	None	4.8% (0–11.6)
		Mild	28.1% (12.4–43.8)
		Severe	67.1% (51.0–83.2)

OR, odds ratio; CI, confidence interval. Estimated marginal means were derived from the generalized linear model (GLM).

## Data Availability

The data that support the findings of this study are available from the corresponding author upon reasonable request. The data are not publicly available due to privacy and ethical restrictions.

## Abbreviations

The following abbreviations are used in this manuscript:

T2DM	Type 2 diabetes mellitus
HbA1c	Hemoglobin A1c (glycated hemoglobin)
FBG	Fasting blood glucose
PSQI	Pittsburgh Sleep Quality Index
OSA	Obstructive sleep apnea
EDS	Excessive daytime sleepiness
GLM	Generalized linear model
IQR	Interquartile range
BF_10_	Bayes factor in favor of the alternative hypothesis
